# Editorial: Five Years of Journal of Cognitive Enhancement

**DOI:** 10.1007/s41465-022-00239-6

**Published:** 2022-03-02

**Authors:** Lorenza S. Colzato

**Affiliations:** Cognitive Neurophysiology, Department of Child and Adolescent Psychiatry, Faculty of Medicine, TU Dresden, Dresden, Germany

Despite the COVID-19 outbreak, we did glance some hint of normality this year. The fifth year of Journal of Cognitive Enhancement (2021) included a broad overview of theory-driven cognitive enhancing articles raging from critical reviews to research articles.

In the issue of March, of exceptional interest is the critical review article by Thiermann and Sheate (2021) about the way to move forward in mindfulness and sustainability. The authors did an admirable job in identifying the leading theoretical links between mindfulness and sustainability, such as “reduced automaticity, enhanced health and subjective well-being, greater connectedness with nature, improved pro-sociality, recognition of intrinsic values, and openness to new experiences” (Thiermann & Sheate, 2021). Notably, the article proposes a research agenda for the potential use of mindfulness to help transform people’s motivations for sustainability.

In the issue of June, the research article by Haslam et al. (2021), testing a very large sample size (*n* = 1328), showed that thinking morally reduced the public’s willingness to optimize cognition overall. Remarkably, promoting moral reasoning exclusively diminishes openness to using brain stimulation among other methods to optimize cognition. I am positive that these findings will spark new investigations to tackle how specific moral concerns drive public hesitancy about brain stimulation.

In the issue of September, a seminal investigation carried out in the lab of Shawn Green (Vodyanyk et al., 2021) addressed the very timely issue that any positive findings in the field of cognitive training are due to placebo effects. In a series of four experiments, the authors cleraly and very convincigly provide evidence against the idea that placebo effects are key elements in triggering positive effects previously attributed to cognitive training interventions.

In the issue of December, the research article by Ørskov et al. (2021) nicely explored individual differences as predictors of performance change during cognitive training. Interestingly, the findings are in line with the magnification account “with higher ability individuals starting out at a higher performance level and showing a higher rate of performance change, and moreover, being more likely to adhere to the training protocol” (Ørskov et al., 2021).

Along the lines of 2021, we will keep looking for interesting special issues; please let us know about any thoughts you might have on these topics.

Let me conclude by thanking all our loyal readers and authors and, of course, all our enthusiastic reviewers, who are so essential for maintaining the journal’s high quality. This will be my final year as Editor in Chief for this journal, which I had the opportunity to proudly found five years ago, as I will start new scientific avenues in the Republic of China. With gratitude, I would like to send you warm wishes for a safe and healthy 2022!
